# Autologous Conditioned Plasma and Hyaluronic Acid Injection for Isolated Grade 4 Osteochondral Lesions of the Knee in Young Active Adults

**DOI:** 10.7759/cureus.27787

**Published:** 2022-08-08

**Authors:** Aysha Rajeev, Mohammed Ali, Kailash Devalia

**Affiliations:** 1 Trauma and Orthopedics, Gateshead Health NHS Foundation Trust, Gateshead, GBR; 2 Trauma and Orthopedics, University Hospital of North Durham, Durham, GBR

**Keywords:** young adults, knee, osteochondral defects, platelet-rich plasma, autologous conditioned plasma

## Abstract

Objectives: To compare the short-term benefits and results of autologous conditioned plasma (ACP) and hyaluronic acid (HA) injection in osteochondral defects in the knee of young adults. The effectiveness of intra-articular platelet-rich plasma (PRP) injections has been evaluated in osteoarthritis. However, few studies investigated its efficacy in knee osteochondral defects.

Methods: This is a retrospective analysis of prospectively collected data. A matched cohort of 30 patients in each group was studied. Group 1 received three HA injections at weekly intervals, and group 2 received three ACP injections at two weekly intervals. We measured Kujala, Lysholm, Oxford, and visual analog scale (VAS) scores at baseline, six, 12, and 36 months to assess function and pain.

Results: Most lesions were in the medial femoral condyles in both groups, followed by lateral femoral condyle and patellofemoral regions. In group 1 (HA), the mean pre-injection scores for Kujala, Lysholm, and Oxford improved significantly at six and 12 months. The scores decreased at 36 months, however, they remained significantly better than the baselines (P < 0.05). The pre-injection VAS scores continued to improve significantly from 6.06±0.785 to 3.40±0.912 at 36 weeks. In group 2 (ACP), VAS and the outcome scores showed a consistent and statistically significant improvement from pre-injection to 36 months.

Conclusions: Our study confirms the short-term clinical benefits of using ACP for symptomatic osteochondral defects of the knee. Further high-quality comparative studies with longer follow-ups are needed to ascertain whether ACP is beneficial in the long term.

## Introduction

Articular cartilage is composed of a dense extracellular matrix (ECM) with a sparse distribution of highly specialized cells called chondrocytes. The ECM is composed of water, collagen, and proteoglycans, with other noncollagenous proteins and glycoproteins in smaller amounts [1]. Articular cartilage provides a smooth, lubricated surface for articulation and facilitates the transmission of loads with a low frictional coefficient [1]. The causes of osteochondral defects in the knee may be due to repetitive trauma, genetic abnormalities, and avascular necrosis [2]. The incidence of knee osteochondral defects is about 65%, as reported in routine arthroscopies [3-5]. It is well documented that articular cartilage is devoid of nerves, blood supply, and lymphatics; hence, most osteochondral defects remain asymptomatic [6]. The inability to regenerate was initially noted by Hunter [7] in 1742, and subsequently, the natural history of these defects and complications, including osteoarthritis and loss of function, were reported. Surgeons, over the years, tried different methods of treatment, including debridement, drilling of the defect, microfracture, and abrasion arthroplasty [7] and, more recently, procedures to restore hyaline or hyaline-like cartilage as osteochondral autograft transplantation [8] and autologous chondrocyte implantation [9,10]. The osteochondral autograft transplantation procedure's disadvantages are the defect's size and donor site morbidity [11,12]. The limitations of the autologous chondrocyte implantation procedure are that it is a two-stage procedure that takes careful and precise planning, cell culture in the labs, and considerable cost implications.

Lately, autologous conditioned plasma (ACP) or platelet-rich plasma (PRP) has become very popular among the orthopedic community as a minimally invasive way of enhancing tissue healing in different conditions, including rotator cuff repair, patellar tendinopathy, knee osteoarthritis, lateral epicondylitis, osteochondral lesions of the talus and other orthopedic conditions [13]. It has been postulated that PRP promotes soft tissue healing by delivering a higher than average concentration of platelets and therefore increases the concentration of platelet-derived growth factors in the diseased area [14]. This has been shown in various studies [13,15]. There are two types of PRP, namely the leukocyte-rich (LR-PRP) and leukocyte-poor (LP-PRP) types. According to previously published evidence, the LR-PRP has better results in tendinopathies, while LP-PRP works better in cases of osteoarthritis [16-18].

The use of PRP in treating osteochondral defects has become more prevalent in recent times. A recently published systematic review by Yausep et al. [19] concluded that PRP, as an adjunct to talus microfracture surgery, significantly improved function and reduced pain compared to microfracture surgery alone. Intra-articular talus PRP injection also demonstrated significantly enhanced recovery of function and decreased pain scores compared to hyaluronic acid (HA). This study aims to compare the short-term benefits and results of ACP and HA injection in osteochondral defects in the knee of young adults.

## Materials and methods

This study was a retrospective matched cohort study that did not require institutional review board (IRB)/ethics committee approval. Data was collected from the operative theatre records, patient notes, arthroscopic examination notes, outpatient clinic letters, and the Picture Archiving and Communication System (PACS) version 6 (Centricity, GE Healthcare, Chicago, USA) for image evaluation. The data were prospectively collected, and outcome scores were routinely collected throughout the follow-up to assess patients' progress. We reviewed the clinical records of all patients who had ACP or HA intra-articular knee injections for osteochondral defects (OCD). The inclusion criteria were that all patients had demonstrable OCD during arthroscopic surgery and received no specific treatment for the lesion, such as debridement or micro-fracture. All included patients had grade 4 OCD according to the International Cartilage Repair Society (ICRS) classification system. The age group was below 45 years, and all patients received treatment in the nature of either HA or ACP injections due to ongoing symptoms. We excluded patients with reported varus and valgus deformities and those with knee flexion less than 90 degrees pre-operatively.

Furthermore, we excluded patients with radiologically evident degenerative changes. All patients completed the baseline functional assessment before receiving the treatment. Group 1 received three HA (Synvisc) injections weekly, and group 2 received three ACP injections at two weekly intervals. The outcome measure scores were then prospectively collected. The follow-up was carried out in the clinic by a senior physiotherapist at six, 12, and 36 months. Kujala, Lysholm, Oxford knee scores, and the visual analog scale (VAS) score were utilized to assess the pain and functional outcomes on every visit.

The ACP was prepared under sterile precautions. An Arthrex double syringe (Arthrex, Naples, FL, USA) was used to draw 15 ml of blood from the vein. The syringe was then placed in an Arthrex centrifuge with a counterweight and centrifuged for five minutes at 1500 revolutions. When the centrifuge was finished, the double syringe was taken out, and the separated plasma was withdrawn from the second syringe. This was injected into the knee joint under sterile conditions after infiltrating the skin with 1% of 5 ml of lignocaine. Statistical analysis was carried out using Statistical Package for Social Sciences (SPSS) version 27 (IBM Corp., Armonk, NY, USA). Paired t-test was used to compare the treatment outcomes at each period for each group separately. Plots and graphs were used to compare the results of the two groups. Significance was set at a p-value less than 0.05.

## Results

Two matching groups of 30 patients were selected after applying the inclusion and exclusion criteria. The mean age of patients in group 1 was 37.8 years and 35.9 in group 2. In both groups males were predominant. Both the left and right sides were affected almost equally in both groups. The most lesions were in the medial femoral condyles in both groups, followed by lateral femoral condyle and patellofemoral. The average size of the lesion in group 1 was 1.32 cm2 and in group 2 was 1.36 cm2. There were no statistically significant differences between the demographics of the two groups (Table 1).

**Table 1:** Patient demographics MFC: Medial femoral condyle, LFC: Lateral femoral condyle, PF: Patellofemoral

Variables		Group 1	Group 2	Significance
Age (mean years)		37.8	35.9	Chi-square tests: P > 0.05
Gender	Male	16	20	
	Female	14	10	
Affected side	Right	14	15	
	Left	16	15	
Location of lesion	MFC	12	14	
	LFC	10	12	
	PF	8	4	
Size of the lesion in cm^2^ (mean)		1.32	1.36	

In group 1 (HA) the mean pre-injection scores for Kujala, Lysholm, and Oxford improved significantly at six and 12 months post-operatively. The scores declined at 36 months although remained significantly better than the baseline. The pre-injection VAS scores continued to improve significantly from 6.06±0.785 to 3.40±0.912 at 36 weeks (Table 2).

**Table 2:** Outcome measure scores for group 1 VAS: Visual analog scale

Outcome scores	Group 1						
	Pre-Injection	6 months		12 months		36 months	
	(mean ±SD)	(mean ±SD)	P value	(mean ±SD	P value	(mean ±SD	P value
Kujala	59.83±2.168	62.67±2.713	<0.001	64.23±2.111	<0.001	63.57±2.889	<0.001
Lysholm	31.67±2.080	33.23±2.112	<0.001	34.67±2.009	<0.001	32.13±2.112	<0.001
Oxford	65.57±2.221	69.77±1.862	<0.001	70.77±1.909	<0.001	66.81±1.811	<0.001
VAS (pain)	6.06±0.785	3.69±1.009	<0.001	3.80±0.884	<0.001	3.40±0.912	<0.001

In group 2 (ACP) all the outcome scores showed a consistent and significant improvement from pre-injection to 36 months (p<0.05). The pre-injection VAS scores also showed a fall from 5.76±0.850 to 3.39±0.915 which is statistically significant (Table 3).

**Table 3:** Outcome measure scores for group 2 VAS: Visual analog scale

Outcome scores	Group 2						
	Pre-Injection	6 months		12 months		36 months	
	(mean ±SD)	(mean ±SD)	P value	(mean ±SD	P value	(mean ±SD	P value
Kujala	58.68±2.968	70.77±2.513	<0.001	73.32±2.111	<0.001	92.13±2.887	<0.001
Lysholm	29.24±2.280	40.28±2.012	<0.001	43.12±2.109	<0.001	45.67±2.012	<0.001
Oxford	62.78±1.921	90.07±1.841	<0.001	92.79±1.299	<0.001	93.51±1.914	<0.001
VAS (pain)	5.76±0.850	3.96±0.909	<0.001	3.82±0.984	<0.111	3.39±0.915	<0.001

**Figure 1:**
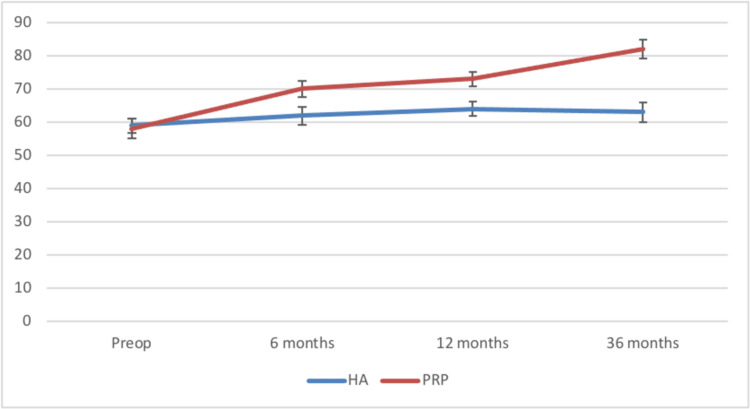
Comparison of the Kujala scores between the two groups HA: Hylauronic acid, PRP: Platelet-rich plasma

The Kujala scores remained unaltered for group 1 but showed a definite improvement at 12 and 36 months for group 2 (Figure 1).

**Figure 2:**
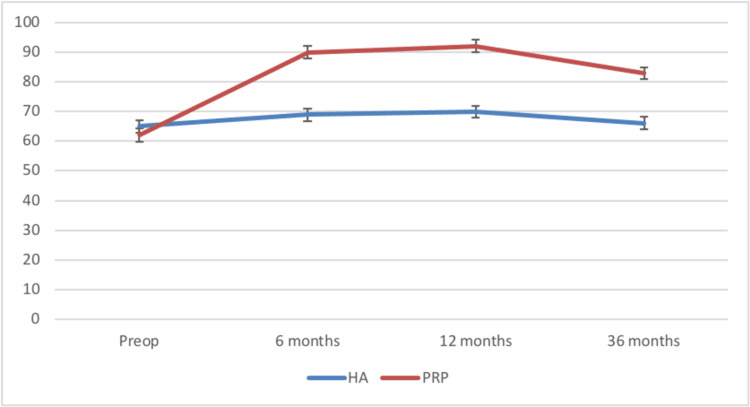
Comparison of the Lysholm scores between the two groups HA: Hylauronic acid, PRP: Platelet-rich plasma

The Lysholm scores for both groups declined at 36 months but for group 2 the fall was comparatively less (Figure 2).

**Figure 3:**
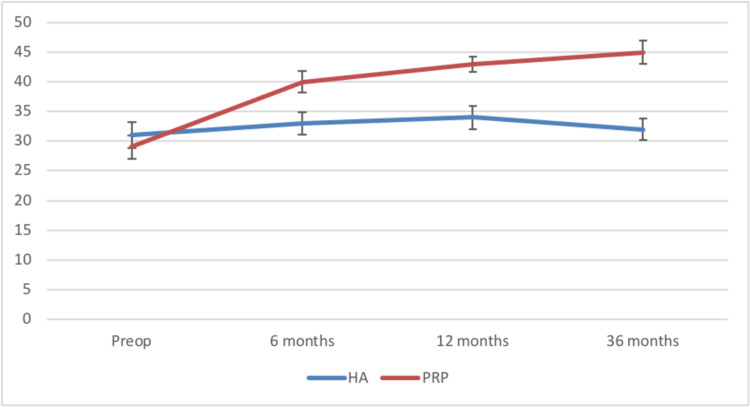
Comparison of the Oxford scores between the two groups HA: Hylauronic acid, PRP: Platelet-rich plasma

The Oxford scores for group 2 showed a significant improvement compared to that of group 1 which remained more or less the same at 36 months (Figure 3).

**Figure 4:**
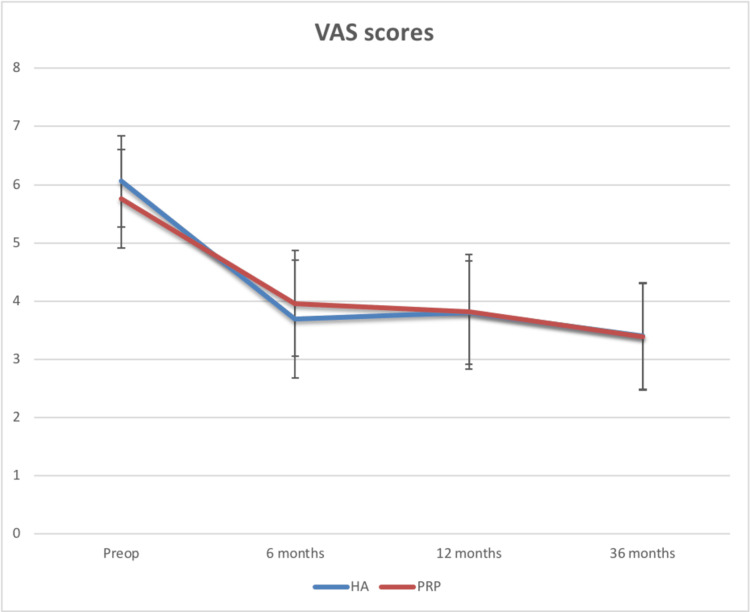
Comparison of the VAS scores between the two groups HA: Hylauronic acid, PRP: Platelet-rich plasma, VAS: Visual analog scale

The VAS scores for pain in both groups improved at the end of 36 months (Figure 4).

The mean differences in the outcome scores for different sites of the lesion in the knee were also studied in the two groups (Tables 4, 5).

**Table 4:** Group 1's comparison of the mean of the outcome scores for different sites of lesion in the knee MFC: Medial femoral condyle, LFC: Lateral femoral condyle, PFJ: Patellofemoral joint, CI: Confidence interval

Outcome scores	Site of lesion											
	MFC				LFC				PFJ			
	Mean difference	95% CI		p-value	Mean difference	95% CI		p-value	Mean difference	95% CI		p-value
		Lower	Upper			Lower	Upper			Lower	Upper	
Kujala	2.167	1.195	3.133	<0.001	3.267	2.153	4.380	<0.001	4.185	2.940	5.430	<0.001
Lysholm	2.111	1.587	2.633	<0.001	2.150	2.100	2.200	<0.001	3.527	2.624	4.430	<0.001
Oxford	2.098	1.412	2.784	<0.001	2.651	1.821	3.480	<0.001	3.426	2.312	4.540	<0.001

**Table 5:** Group 2's comparison of the mean of the outcome scores for different sites of lesion in the knee MFC: Medial femoral condyle, LFC: Lateral femoral condyle, PFJ: Patellofemoral joint, CI: Confidence interval

Outcome scores	Site of lesion											
	MFC				LFC				PFJ			
	Mean difference	95% CI		p-value	Mean difference	95% CI		p-value	Mean difference	95% CI		p-value
		Lower	Upper			Lower	Upper			Lower	Upper	
Kujala	4.797	4.182	5.412	<0.001	4.261	3.100	5.421	<0.001	2.694	1.964	3.422	<0.001
Lysholm	4.280	3.012	5.548	<0.001	4.115	2.988	5.241	<0.001	2.735	1.826	3.644	<0.001
Oxford	4.474	3.294	5.654	<0.001	3.900	2.812	4.988	<0.001	2.648	1.714	3.582	<0.001

There is a significant mean difference in the scores for medial femoral condyle (MFC) and lateral femoral condyle (LFC) compared to that of the patellofemoral joint (PFJ) between pre-injection and at 36 months (p<0.001). Both the HA and ACP groups showed that the mean outcome scores were low for PFJ and better scores were achieved for MFC. None of these patients developed any complications. 

## Discussion

Articular cartilage injuries and defects are among the most common causes of morbidity in active young adults, affecting hip, knee, or ankle joints [15]. The role of the new biological treatments in orthopedics, such as PRP, has remained controversial, especially when dealing with osteochondral defects as they cannot regenerate [20]. Hence, the effects of PRP on chondrocytes have been studied thoroughly both in vitro and in vivo, and part of these studies concluded that PRP increases the synthesis of chondrocytes. Furthermore, the published evidence highlighted many ways of preparing PRP such that the concentrations of platelets and leukocytes can vary considerably. We noted that in some in vitro studies, LR-PRP promoted acute inflammatory responses and increased synoviocyte death [21], whereas LP-PRP stimulated chondrocytes to express type II collagen and aggrecan [22]. Notably, the double syringe system used in our study generated LP-PRP, which Noh et al. [23] reported has higher transforming growth factor beta 1 (TGF-b1) and fibroblast growth factor 2 (FGF-2) concentrations than leukocyte-rich PRP produced with the GPS III Platelet Concentration System (Zimmer Biomet, Warsaw, IN, USA). Khurana et al. compared the efficacy of PRP with ACP for early knee osteoarthritis at six months follow-ups and concluded that there was no difference between the two injections [24].

Few reported studies looked at the efficacy of PRP in treating cartilage injuries. Sanchez et al. [25] conducted an observational retrospective cohort study using intraarticular injections using autologous preparation rich in growth factors (PRGF) and found that the outcome scores and pain are much better after five weeks of osteoarthritis of the knee. Filrado et al. [26], in their study with intra-articular platelet-rich plasma injections, found that at the end of 12 months, PRP injections can reduce pain and improve knee function and quality of life in young patients with articular cartilage degeneration. A recent systematic review by Elghawy et al. [27] concluded that PRP might show clinical benefit in those with osteochondral lesions of the talus in terms of pain and functionality, although chondral regeneration via MRI is inconsistent. Shi et al. in 2017 [28] conducted a systematic review to evaluate the clinical results of PRP and mesenchymal stem cell treatments (MSC) for articular cartilage lesions and knee osteoarthritis. Thirty-three articles were included in Shi et al.'s study. Out of which, the PRP was utilized in 21 studies. All PRP studies showed improved pain and functional outcomes. Two studies reported no significant difference in improvement compared to HA [28].

Our study revealed that PRP and HA significantly improved patients’ functional outcomes. Those who had ACP achieved better results than the HA group, and the effect lasted for 36 months; both groups remained better than their baseline. Compared to published literature by Varun et al. [29], our study targeted a younger population with a mean age of 36.87 years between the two groups. In addition, the ACP has been proven safe with no significant side effects when injected intra-articularly. Furthermore, it is the patient’s blood, and this eliminates the risk of blood-prone infections. The ACP can be injected regularly to improve function and symptoms for young patients not old enough for joint replacement and elderly patients not fit for surgery.

The study is limited by its retrospective nature and the relatively small number of patients. We could have tested the effect of different variables on treatment, such as age, gender, and body mass index. Additionally, no radiological assessment was done following the injections to examine the healing outcomes. On the other hand, the study has many strengths, including the adequate follow-up duration and using multiple outcome scores to assess the efficacy of the ACP vs HA.

## Conclusions

Even though ACP is gaining popularity as a treatment for osteochondral lesions, few studies have been published that have sufficient clinical data on functional outcomes, large sample sizes, prolonged follow-up periods, and a thorough explanation of the molecular and cellular mechanisms of ACP action. Additionally, rigorous methods for ACP preparation, standardization, and patient post-treatment management must be developed. The aforementioned points to the need for additional clinical and fundamental scientific research to address these objectives. Our study suggests short-term clinical benefits with ACP use for symptomatic osteochondral defects of the knee. This procedure has proven to be safe when done under sterile precautions. The two groups improved significantly following treatment, however, the ACP group achieved better and more consistent results than the HA group. Further high-quality comparative studies with longer follow-ups are needed to ascertain whether PRP is beneficial in the long term.
